# Selfhood-attribution in a social context: further evidence for a Pars-Pro-Toto account

**DOI:** 10.3389/fpsyg.2025.1528172

**Published:** 2025-10-03

**Authors:** Jan Pohl, Kristina Nikolovska, Francesco Maurelli, Arvid Kappas, Bernhard Hommel

**Affiliations:** ^1^Faculty of Psychology, Dresden University of Technology, Dresden, Germany; ^2^Adaptive Systems Group, Department of Computer Science, Humboldt University of Berlin, Berlin, Germany; ^3^School of Computer Science and Engineering, Constructor University, Bremen, Germany; ^4^School of Business, Social and Decision Sciences, Constructor University, Bremen, Germany; ^5^Shandong Provincial Key Laboratory of Brain Science and Mental Health, Faculty of Psychology, Shandong Normal University, Jinan, China

**Keywords:** self, mind, agency, attribution, human-robot interaction, non-humanoid robot, social

## Abstract

**Introduction:**

Humans show a consistent tendency to anthropomorphize or attribute aspects of selfhood to non-human agents. In a previous study, we found that people (over-)generalize from the presence of a single behavioral selfhood cue (like equifinality or efficiency) to the presence of other (actually absent) cues, suggesting that a small aspect of selfhood suffices to activate the entire selfhood concept with all its other implications (Pars-Pro-Toto).

**Method:**

Our previous study was exclusively manipulating non-social aspects of selfhood. However, the contribution of social interaction in developing a concept of “Self” has been stressed in the literature. Thus, in the present study we tested whether these findings can also be demonstrated for social aspects. Specifically, we manipulated the presence or absence of cues indicating social sensitivity, attention sharing, or helping behavior in small non-humanoid robots, and tested which cues would elicit attributions of various aspects of selfhood.

**Results:**

The results replicated our previous finding that the presence of a single cue is sufficient to (over-)generalize to other, non-manipulated cues, extended our previous observations to social conditions and provided further support for our Pars-Pro-Toto account. It is noteworthy that participants showed a stronger tendency to overgeneralize to other social selfhood-related characteristics than to the non-social characteristics. Moreover, compared to our previous study, participants no longer showed a consistently stronger attribution of agency to the robot that was exhibiting cues for one of the critical characteristics.

**Discussion:**

The missing effects of agency are discussed as reflecting how sociality might be construed vis-à-vis individual goal pursuit. The interplay between sociality and individuality might be linked to our perception of agency in other agents when these are part of a group. The results are further discussed in light of the increasing presence of robots and other artificial agents in everyday life, as they support a shift in focus from their actual capabilities toward what people's expectations of these systems are. We stress that it is important to consider technological systems in their social relation to people as they tend to attribute complex concepts such as selfhood even when only perceiving simple behavioral cues.

## 1 Introduction

Humans have a strong tendency to attribute humanlike aspects of selfhood to nonhuman agents and to treat them accordingly. For example, the Media Equation illustrates that people interact with technological agents the same way (i.e., applying social norms) as they would with humans (Nass et al., 1999; Reeves and Nass, 1996). Moreover, people ascribe personality to dogs (Gosling et al., 2003), and intent to automatic doors (Ju and Takayama, 2009) or even to nonrandom motion (Heider and Simmel, 1944). Further, humans have unique capabilities in explaining others' behavior based on their assumed mental states, such as believes and intentions (see e.g., Theory of Mind, Frith and Frith, 2005), as well as predict others' behavior (see e.g., Intentional Stance, Dennett, 1989). Yet, the scientific debate on defining the “self” is still ongoing (Leary, 2004; see also Gallagher, 2013, for an overview of theories), and the argument has even been raised that such a thing as an objective self may not exist (e.g., Bennett and Hacker, 2022; Giles, 1993). Therefore, in the present study we are not investigating “the self,” rather, it is the subjective idea of a self that laypeople have in everyday life and that they tend to attribute to other agents. Irrespective of what people's inferences about others contain and irrespective of an individual's concrete concept of what the self is, the question arises as to what causes this attribution in people and how it functions.

Heider's (1958) attribution theory tries to answer this with the concept of “naïve realism”. He claims that people are typically not aware of the subjective nature of their perception of the world, they assume the ambiguous perceptual information to be objective (see also Cheek et al., 2021; Pronin and Hazel, 2023) and form theories explaining others' behavior often based on the misconception that actions are a result of personal causes like agency and intention (see also fundamental attribution error, Ross, 1977). Similarly, Brunswik (1955) assumes that people do not have a direct access to the objective state of the world. In the development of his lens model he claims that the world is complex and uncertain and what people perceive are only proximal cues of the environment. People have to infer the objective world from these cues that only have a probabilistic information about the state. For selfhood, this implies that people may utilize behavioral cues to derive a judgement on whether some observed agent has a self. We emphasize that our study was not motivated by any particular scientific definition of what a self may or may not be, nor do we assume that our findings support some definitions more than others. Instead, we were interested in the way naïve people would implicitly define and attribute selfhood as well as how they would deal with this concept in their interactions with artificial agents. Given our agnostic approach to existing scientific definitions, we also did not make any attempt to assess or test whether the naïve selfhood concept under investigation would be “correct” or “appropriate.”

**Figure 1:**
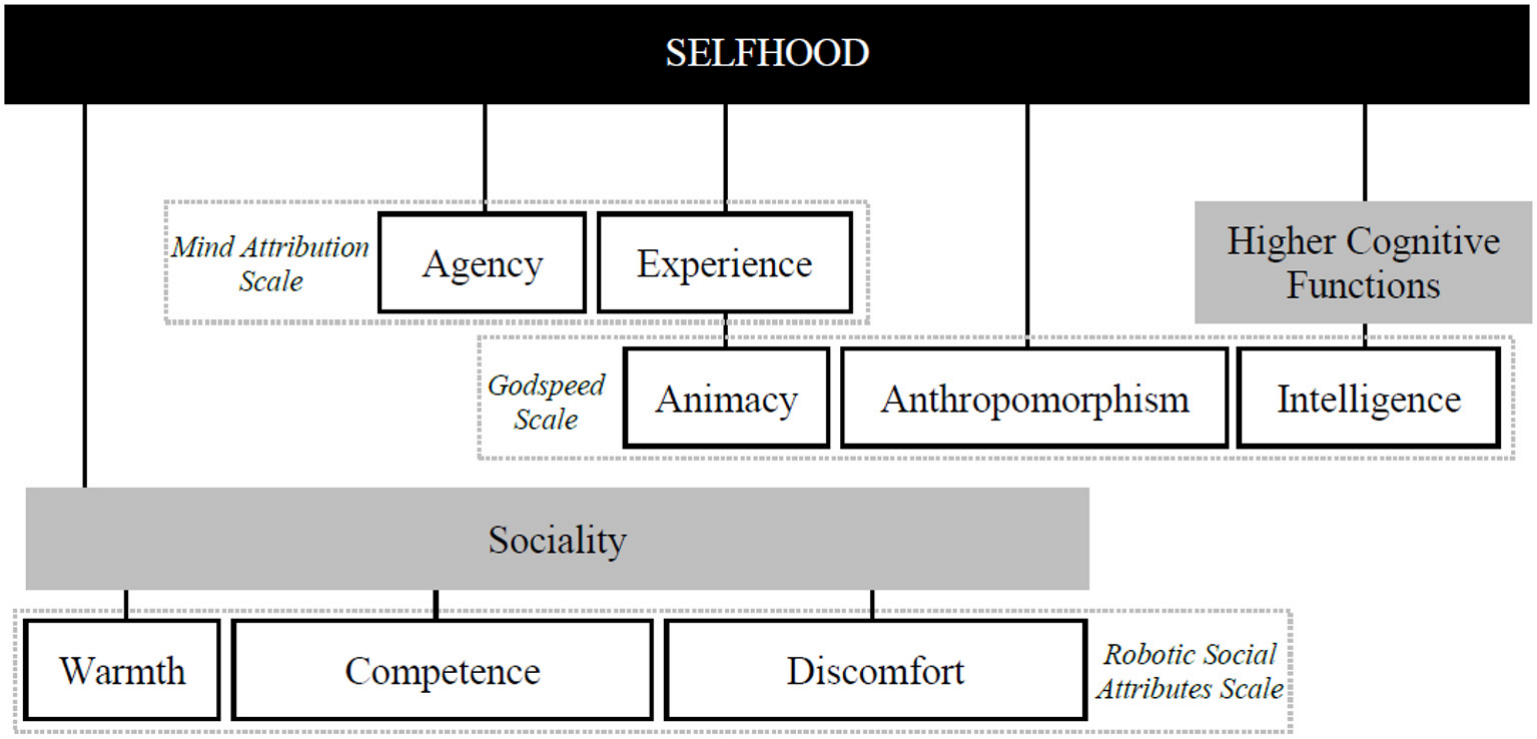
Measurements for different facets of selfhood. Overview of instruments used to access selfhood ratings via a broad spectrum of related concepts. This operationalization enables to be agnostic about specific theories of the self and whether it exists or not. The Godspeed Scale includes also the subscales perceived safety and likeability, which were also implemented in the studies but were not considered critical for selfhood-attribution.

In a previous study, we employed the same theoretical approach to identify the criteria that people might use for attribution of a “Self” in a moving object, and the behavioral cues signaling such criteria (Pohl et al., 2024, under review). To this aim, we manipulated the behavior of non-humanoid, simple robots (similar to a vacuum cleaning robot) to suggest either the presence (C+ or critical) or absence (C− or control) of a potential core characteristic to support self-attribution. Participants were presented with videos of a single robot navigating though abstract obstacles (white and black cubes) in ways that either did reflect the particular characteristic (e.g., by navigating in an efficient fashion; C+) or not (navigating inefficiently, C−). Participants were to rate robots under C+ and C− conditions on a scale about the behavioral characteristics (also functioning as a manipulation check) and several established questionnaires about selfhood-related factors, such as agency, experience, anthropomorphism and intelligence (see Figure 1 for the specific questionnaires used). As we remain agnostic to whether the self exists or which definition would be most accurate we chose this novel operationalization to have a broad range of relevant dimensions considering selfhood without having to commit to any definition of selfhood. We believe that selfhood is a suitable candidate as an umbrella term for these dimensions, as they may originate from that subjective first-person perspective that is experiencing something that is then labeled as having a self, a mind, being conscious, intelligent, or social. As such we do not feel that it is possible to provide a definite definition of what we deem selfhood, we see it as a highly subjective concept. This does not imply that such a thing as an objective self does not exist, but, on the other hand, the subjective experience of a sense of selfhood and the attribution of this concept also do not imply that it has to exist.

In our previous study we investigated several potential core characteristics in separate experiments: causality, equifinality, behavioral efficiency, as well as learning and context sensitivity. As expected, participants attributed the manipulated characteristic to the critical robot in almost all experiments along with significantly higher ratings on the self-related scales than the control robot. Unexpectedly, however, most of the investigated characteristics boosted the scores of other, non-manipulated, characteristics as well (e.g., considering a more efficiently acting robot as also showing more causal behavior, even if the latter was not manipulated), suggesting that participants systematically over-generalized selfhood-relevant behavioral cues. We concluded attributing selfhood does not rely on 1:1 associations between perceived behavioral cues and internal representations of the respective characteristics. Rather, one given cue can activate multiple representations even without clear-cut perceptual evidence for the represented feature. One available aspect of selfhood seems to imply the presence of other, currently unavailable aspects—an assumption that we coined a *Pars-Pro-Toto* account.

An important limitation of our previous study was that all agents that we presented to participants were acting on their own, in the absence of any social interaction, so none of our investigated characteristics was social in nature. However, many authors have emphasized the importance of social interaction to the emergence and functions of a self (e.g., Cooley, 1998; Mead, 1913), which might suggest that (real or implied) social interaction represents a crucial factor in attributing selfhood to other agents. Accordingly, we were interested in testing whether our previous observations would generalize to social characteristics as potential core characteristics in selfhood-attribution. In the present study, we considered three possible characteristics: *social sensitivity*, the ability to consider the presence and interests of others when following one's own goals; *attention sharing* is an important feature of social sensitivity found in humans and non-human animals alike—ranging from following other agent's gaze to enabling seeing-knowing relationships (see, Moore and Dunham, 1995). Thus, this characteristic can be understood as a reaction to internals states of other agents and as such attention sharing seems a likely candidate for core characteristics in the attribution of self-hood. Lastly, *helping*, a more direct and overt form of social interaction that might represent a uniquely human trait (Warneken and Tomasello, 2006). While this is not a complete list of social characteristics humans show while interacting with each other, they feature a broad range suitable for extending our previous manipulations into the social domain. We manipulated these three behavioral characteristics in three separate experiments (Experiment 1, 2, and 3, respectively), with methods that were closely following the procedure of our previous study, except that a few scales were added to the dependent variables assess the social aspects of the study. Our key question was whether the previously observed Pars-Pro-Toto effect (over-generalization from manipulated to other, not-manipulated self-related characteristics) could also be demonstrated for social characteristics and whether this over-generalization would be limited to other social characteristics or also apply to non-social characteristics, like equifinality or behavioral efficiency.

## 2 Method

The key difference to our previous study (Pohl et al., 2024, under review) was the addition of a second robot that our robot of interest was able to interact with. Accordingly, the scale accessing the perception of the behavioral characteristics was extended to items reflecting the new characteristics (scales and other materials can be found at the Open Science Framework [OSF; Foster and Deardorff (2017)] under https://osf.io/cqf6d/). We also added the complete Robotic Social Attributes Scale (Carpinella et al., 2017) and two questionnaires collecting participant-related data, to be filled in at the end of the session.

### 2.1 Participants

Participants were recruited from the UK via Prolific. Recruitment continued until 80 valid data sets were collected for each of the three experiments; see Table 1 for demographic data. Participants received £4 for completing the study. Participants were excluded from the analysis if they reported considerable technical difficulties (e.g., stuttering of the videos, which resulted in the exclusion of *n* = 14 or 5% of the total number of recruited participants) or if they failed the attention check (as described in the *Procedure* section below, which resulted in the exclusion of *n* = 8 or 3% of all recruited participants).

**Table 1:** Demographic overview of participants per experiment.

			**Age**		**Gender (in %)**		**PERQ** ^*****a*****^		**AQ** ^*****b*****^	
**Exp**.	***n*_*total*_**	***n*_*included*_**	***M***	***SD***	**Female**	**Male**	***M***	***SD***	***M***	***SD***
1	88	80	39.88	11.70	49	51	1.99	0.70	3.12	2.06
2	84	80	41.66	14.64	49	51	2.00	0.75	3.20	2.44
3	90	80	38.99	13.53	51	49	2.00	0.76	3.21	1.87

^a^ Data from Item 4 of the Prior Experience with Robots Questionnaire (PERQ), which is a self-reported knowledge estimate about robots and their capabilities on a scale of 1 to 5. ^b^ Score of the Autism Spectrum Quotient (AQ) AQ-10 with a range of 0 to 10, where 6 is the recommended threshold for refering to a specialist diagnostic assessment.

### 2.2 Material

#### 2.2.1 Stimuli

Stimuli consisted of videos captured using a Panasonic HC−V380EG-K camera in our laboratory. They were shot from above at an angle showing a “Duckie Mobile Bot” (Paull et al., 2017) robot identified by either a white triangle or square and a second unmarked robot. The marked robots were the robots of interest to be rated by the participants. We also utilized white and black cardboard cubes as obstacles in all experiments (the stimuli can also be found in the online material at https://osf.io/cqf6d/).

The robots of interest were remotely controlled to cue either the presence (C+ or critical) or absence (C− or control condition) of a critical characteristic. The other robot was either also remotely controlled or pre-programmed. Each experiment presented an equal number of stimuli for C+ and C−. Additionally, there was always a pair of a video showing the C+ and a video showing the C− robot in the same setup with the same duration, but between pairs the video duration was not the same in all experiments (between 8 s and 17 s).

In *Experiment 1*, to manipulate the presence vs. absence of perceived social sensitivity, 2 sets of 5 videos with a duration of 8 s to 11 s were recorded. In the videos of both conditions the marked robot moved from one side of the screen to the other while circumventing obstacles. Those obstacles were multiple cubes and another unmarked robot (moving or stationary). In the C+ stimuli videos, the critical robot suggested awareness of the other robot by turning in a way similar to paying attention to the other robot, whereas in the C− stimuli, the control robot simply moved around the other one if necessary and otherwise it just ignored the other one. Across stimuli pairs the second unmarked robot showed different behaviors, in one pair it was stationary, while in another, it moved in circles, and in the 3 other stimuli sets it showed an oscillating movement.

In *Experiment 2*, to manipulate attention sharing, two sets of 5 videos with a duration of 3 s to 9 s were recorded. In all videos the second unmarked robot showed no movement except a turning move once the marked robot was next to it. In the C+ stimuli videos, the C+ robot first moved to a position close to the unmarked robot unless the starting position of the robots was already next to each other. Once the unmarked robot turned, to suggest looking at an object (a black or white cube, or a formation of multiple cubes), the critical robot also turned in a way to suggest looking at the same object. The videos of the C− stimuli looked almost identical, except that the C− robot turned in a way to suggest looking at a different or no object at all.

In *Experiment 3*, to manipulate helping behavior, 2 sets of 5 videos with a duration of 15 s to 17 s were recorded. In all videos the second unmarked robot moved in a way to suggest that it was trying to move a specific cube with the apparent goal to either (1) get to the other side of cubes arranged in a blockade or (2) simply to move the cube to a desired position, which was not possible in both cases due to the arrangement of the cubes and the manipulation that only black cubes were movable. In videos of both C+ and C− stimuli, at the end of the video the robot of interest returned to its starting position. In cases (1) the C+ robot moved the black cube in view of the other robot, enabling the other one to get to the other side, while the C− robot moved a black cube outside of the other robots viewing field. In cases (2) the C+ robot first tried to move the cube blocking the cube being pushed by the other robot, which was not possible. Then the C+ robot moved the cube that the other robot continues to push to the side so that it was no longer blocked. While the C− robot also first tried to move the blocking cube, it then moved another cube leaving the other robot still unable to move its chosen cube.

#### 2.2.2 Implementation

The study was run as in-browser experiments that participants could participate in from home. The experiments were programmed with jsPsych 7.3 (de Leeuw et al., 2023) and hosted on a university server with JATOS 3.7.4 (Lange et al., 2015).

### 2.3 Procedure

The procedure was the same for all three experiments. They commenced with a consent form and instructions (with approval from Constructor University's Ethics Committee). The experiments then continued with the presentation of stimuli. C+ and C− stimuli were presented in alternation. The videos of one condition were consistently displayed either on the left or the right of the window, while the mapping of condition and alignment was systematically counterbalanced. This was done to ensure that participants knew which robot was presented on any given trial as their physical appearance only differed in a small white marking in the shape of either a triangle or square. Following the stimulus presentation, participants completed several questionnaires. In the first four questionnaires they rated the robots of each condition separately (participants had to answer all questionnaires first for one. then for the other robot), while the subsequent two questionnaires asked about the participants themselves. At the end of each experiment, participants were debriefed, asked what they thought the research question was and whether they experienced any technical difficulties with the study. Those participants who reported major problems (e.g., faulty stimulus presentation) were removed from the analysis (resulting in *n* = 14 exclusions, or 5% of all recruited participants).

The study employed a manipulation check scale (MCS) with three items for each characteristic of interest^1^. E.g., for the characteristic helping the items “It appeared to be helping others,” “It appeared to understand when others needed aid” and “It seemed to seek ways in which it could help others” were used. The items were composed to range from simple descriptions devoid of selfhood-related states to descriptions referencing intentions similar to the answers of participants in Heider and Simmel's study (see the online materials at OSF for the complete questionnaire).

For accessing selfhood-attribution three already established questionnaires were used: The subscales agency and experience of the Mind-attribution Scale (MAS) (Bigman and Gray, 2018), the Godspeed Scale (GS) (Bartneck et al., 2009), as well as the Robotic Social Attributes Scale (RoSAS) (Carpinella et al., 2017). The scales were implemented as continuous scales, ranging from 0 to 100, with sliders marked only at the extremes: 0 and 100 for the MCS; “Disagree” and “Agree” for the MAS; negative and positive items for the GS and RoSAS. An attention check item asking if the robot was able to move supplemented the MAS. As the robots consistently moved in the videos, any participant who moved the response slider toward “Disagree” was excluded from the analysis (resulting in *n* = 8 exclusions, or 3% of all recruited participants). The MCS was completed by all participants first, and the order of MAS, GS, and RoSAS was counterbalanced with the MAS always in the middle to ensure that the attention check was placed at the same point in the study flow.

Further, we used as exploratory measures the Prior Experience with Robots Questionnaire (PERQ) (Schaefer, 2016) and the Autism Spectrum Quotient (AQ) AQ-10 (Allison et al., 2012) to gather additional information about our participants. The PERQ provides information about participants' knowledge about robots and their capabilities while the AQ indicates whether participants may be on the autism spectrum. To avoid participants being influenced by these questionnaires, they were collected after the main experiment.

### 2.4 Data analysis

All statistical analyses were conducted using R (Version 4.2.2., R Core Team, 2022). The analysis code is published in the OSF project. For each experiment, we first analyzed the data of the behavioral characteristics (manipulation check). Here ratings were aggregated by participant, cue-presence (C+ or present vs. C− or absent) and characteristic using the mean. In the analysis we conducted a two-way ANOVA with the within-participant factors of cue-presence (C+ vs. C−) and characteristic (causality, speed, equifinality, efficiency, learning sensitivity, and context sensitivity). If there was a significant interaction, we calculated *post-hoc* paired *t*-tests for cue-presence grouped by characteristic.

Next, in cases of a significant interaction between cue-presence and characteristic, we conducted a separate analysis of the selfhood-attribution data for those experiments. For this analysis we categorized in subscales rather than the overall questionnaires. The data from the MAS was therefore categorized into either agency or experience, while each rating from the GS was categorized as animacy, antropomorphism, likability, perceived intelligence, or perceived safety and RoSAS items were categorized as warmth, competence or discomfort. Ratings were then aggregated by participant, cue-presence, and subscale using the mean. This way we calculated a two-way ANOVA with cue-presence (C+ vs. C−) and subscales (agency, experience, anthropomorphism, animacy, likeability, intelligence, safety, warmth, competence, and discomfort) as within-participant factors. If there was a significant interaction, we conducted *post-hoc* paired *t*-tests for cue-presence grouped by subscale. While we included likeability and perceived safety in the analysis, we did not consider these subscales as significant contributors to accessing selfhood-attribution, rather as scales of interest in the context of social interaction or in general.

## 3 Results

### 3.1 Experiment 1: social sensitivity

**Figure 2:**
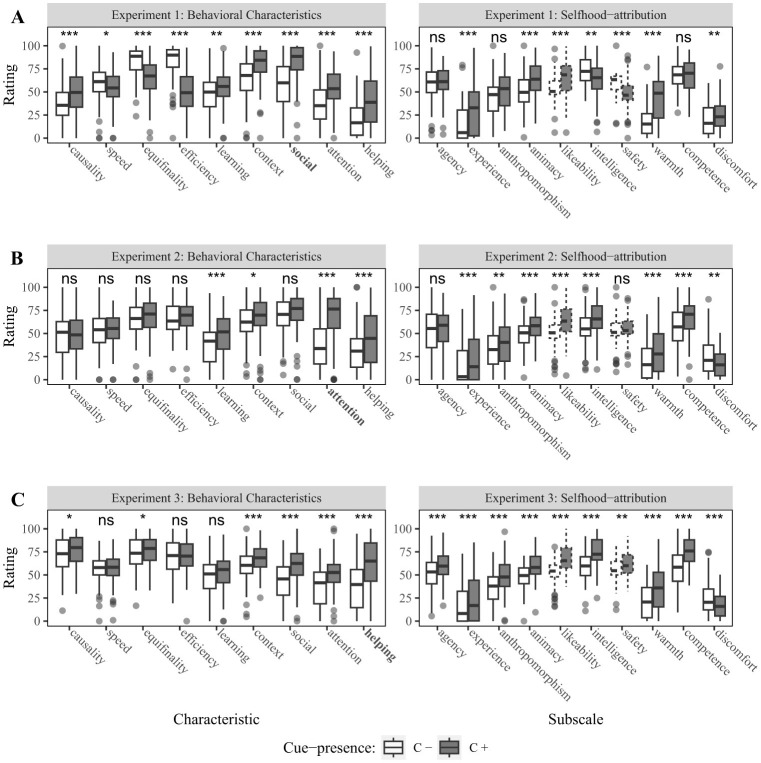
Barplots showing ratings for the behavioral characteristics and selfhood-attribution across experiments. Left side shows results for the behavioral characteristics from our manipulation check scale, with the manipulated characteristic highlighted in bold. The right side shows results for subscales from Mind Attribution Scale (agency and experience), Godspeed Scale (anthropomorphism, animacy, likeability, intelligence and safety) and Robotic Social Attributes Scale (warmth, competence and discomfort). Barplots printed with dotted lines are not considered critical for selfhood-attribution. **(A)** Results from Experiment 1 investigating social sensitivity. **(B)** Results from Experiment 2 manipulating attention sharing. **(C)** Results from Experiment 3 investigating helping behavior. Significance codes: *p* < 0.050 *, *p* < 0.010 **, *p* < 0.001 ***.

For the behavioral characteristics, the ANOVA revealed significant main effects of cue [*F*_(1, 79)_= 4.12, *p* = 0.046, η^2^ = 0.01] and characteristic [*F*_(4.75, 375.18)_= 91.48, *p* < 0.001, η^2^ = 0.30], and a significant interaction [*F*_(3.93, 310.61)_ = 56.38, *p* < 0.001, η^2^ = 0.16]. *Post-hoc* paired *t*-tests showed significant differences for all characteristics with the C+ robot receiving higher ratings for all characteristics except speed, equifinality and efficiency (see, Figure 2A left).

The analysis of the selfhood-attribution data also showed significant main effects of cue-presence [*F*_(1, 79)_ = 10.45, *p* = 0.002, η^2^ = 0.02] and subscale [*F*_(4.08, 322.29)_ = 171.38, *p* < 0.001, η^2^ = 0.45], and a significant interaction [*F*_(4.92, 388.54)_ = 25.68, *p* < 0.001, η^2^ = 0.07]. *Post-hoc* paired *t*-tests further revealed significant differences for all subscales except agency [*t*(79) = 0.05, *p* = 0.958], anthropomorphism [*t*(79) = 1.68, *p* = 0.098] and competence [*t*(79) = 0.08, *p* = 0.937] (all other *p*-values <= 0.007). Ratings were higher for the C− robot on all significant subscales except intelligence (see, Figure 2A right).

### 3.2 Experiment 2: attention sharing

For the behavioral characteristics, we observed a significant main effect of cue-presence [*F*_(1, 79)_ = 20.36, *p* < 0.001, η^2^ = 0.03] and characteristic [*F*_(5.27, 416.65)_ = 55.00, *p* < 0.001, η^2^ = 0.20], and a significant interaction [*F*_(5, 395.31)_ = 22.37, *p* < 0.001, η^2^ = 0.04]. *Post-hoc* paired *t*-tests showed significant differences for the characteristics learning [*t*(79) = 4.07, *p* < 0.001] and context sensitivity [*t*(79) = 2.52, *p* = 0.014], attention sharing [*t*(79) = 7.73, *p* < 0.001] and helping [*t*(79) = 4.08, *p* < 0.001]. The C+ robot was rated higher than the C− robot on these characteristics (see, Figure 2B left).

Next, the analysis of the selfhood-attribution data showed significant main effects of cue-presence [*F*_(1, 79)_ = 27.98, *p* < 0.001, η^2^ = 0.03] and subscale [*F*_(4.35, 343.76)_ = 130.18, *p* < 0.001, η^2^ = 0.39], and a significant interaction [*F*_(3.82, 301.84)_ = 9.04, *p* < 0.001, η^2^ = 0.02]. *Post-hoc* tests revealed significant differences in all subscales except agency [*t*(79) = 1.36, *p* = 0.178] and perceived safety [*t*(79) = 1.23, *p* = 0.221] (all other *p*-values <= 0.006). The ratings for the C+ robot were higher on all subscales except for discomfort (see, Figure 2B right).

### 3.3 Experiment 3: helping

First, for the behavioral characteristics we observed significant main effects of both cue-presence [*F*_(1, 79)_ = 31.65, *p* < 0.001, η^2^ = 0.04] and characteristic [*F*_(4.27, 337.51)_ = 55.51, *p* < 0.001, η^2^ = 0.24], and a significant interaction [*F*_(3.97, 313.56)_ = 20.57, *p* < 0.001, η^2^ = 0.04]. Almost all *post-hoc* paired *t*-tests were significant except for speed [*t*(79) = 0.18, *p* = 0.860], behavioral efficiency [*t*(79) = 0.59, *p* = 0.554] and learning sensitivity [*t*(79) = 1.61, *p* = 0.112] (all other *p*-values <= 0.050). On the significant comparisons the C+ robot was always rated higher than the C− robot (see, Figure 2C left).

Next, the ANOVA of the selfhood-attribution revealed significant main effects of cue-presence [*F*_(1, 79)_ = 51.01, *p* < 0.001, η^2^ = 0.08] and subscales [*F*_(3.90, 307.75)_ = 165.53, *p* < 0.001, η^2^ = 0.51], and a significant interaction [*F*_(3.51, 277.49)_ = 18.11, *p* < 0.001, η^2^ = 0.04]. *Post-hoc* tests showed significant differences on all of the tested subscales (all *p*-values <= 0.003) with ratings for the C+ robot always higher than the C− robot, except for the subscale discomfort—where the C− robot (*M* = 23.99, *SD* = 25.42) received higher ratings than the C+ robot (*M* = 17.22, *SD* = 20.72) (see, Figure 2C right).

### 3.4 Joint analysis

**Figure 3:**
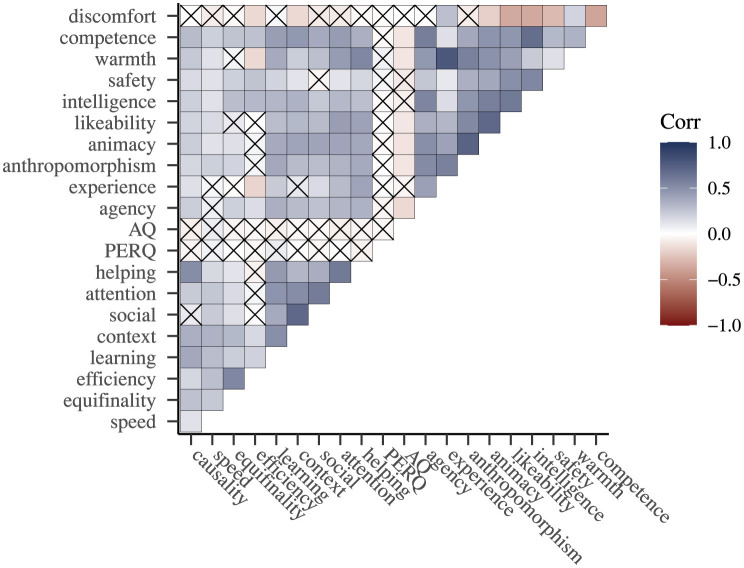
Correlation plot across all experiments. Crossed out correlations are not significant (*p* >= 0.050).

To identify commonalities across the 3 experiments, we calculated Pearson's correlation coefficients for ratings of the characteristics and selfhood-attribution subscales across all experiments. This analysis showed that a majority of characteristics and subscales have significant, positive correlations (63% of all correlation-coefficients), with moderate coefficients accounting for 23% and strong coefficients accounting for 14% of all coefficients (see, Figure 3 for a visual representation).

Note that for the subscale discomfort almost all of the significant correlations are negative, which makes sense as this is the only negatively framed subscale. This subscale further shows an over proportionate number of non-significant correlations suggesting that it is not a crucial dimension for selfhood-attribution.

Next, to characterize the results of the agency attribution we calculated Bayes factors testing for an effect of cue presence on agency using the r package “BayesFactor” (Morey and Rouder, 2024) and following interpretations guidelines as outlined by Kass and Raftery (1995). This was done for each experiment presented here and for the experiments presented in our previous paper (Pohl et al., 2024, under review).

For the social experiments, this analysis reveals decisive evidence for an effect of cue-presence on agency for the last experiment (helping, *BF*_10_ > 100) and for the first two experiments no evidence for an effect of agency (social sensitivity: *BF*_10_ = 0.05; attention sharing: *BF*_10_ = 0.44) and in the case of social sensitivity strong evidence against such an effect (*BF*_01_ = 19.41).

For the non-social experiments, we found decisive evidence for an effect of cue-presence on agency for the experiments manipulating causality (*BF*_10_ > 100), equifinality (*BF*_10_ > 100) and learning sensitivity (*BF*_10_ > 100) as well as strong evidence for the experiment manipulating efficiency (*BF*_10_ = 40.70). Surprisingly, even for the last experiment (context sensitivity), where the manipulation check did not reveal any significant differences in the perception of the characteristics, we found substantial evidence (*BF*_10_ = 9.19) for an effect of cue-presence on agency.

### 3.5 Exploratory analysis

**Figure 4:**
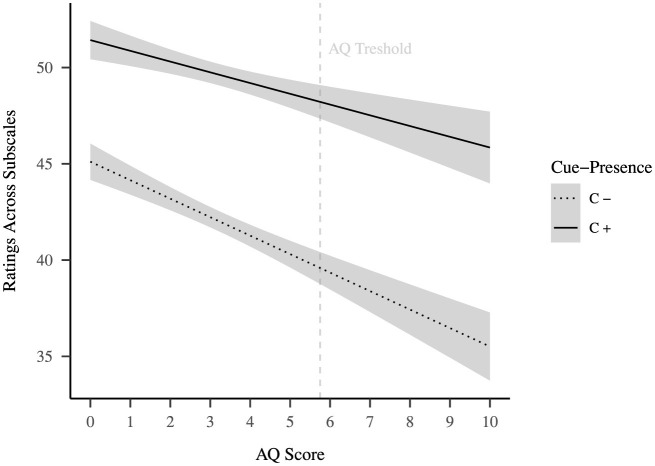
Exploration of selfhood-attribution in relation to autism quotient. In light gray a GLM-fit of ratings across all selfhood-related subscales is shown separated by cue-presence. Scoring 6 or higher on the AQ-10 is an indication for autism (it is not a diagnosis).

Since the situations presented in our experiments were social in nature we additionally investigated whether participants' attribution toward the robots was modulated by autism. For this purposes we calculated a three-way ANOVA with within-subjects factors cue-presence and subscale, as well as the between-subject factor Autism Spectrum Quotient (AQ). The ANOVA revealed a significant main effect of the AQ [*F*_(1, 25, 400)_ = 99.93, *p* = 0.004], with overall lower ratings with higher scores (see, Figure 4). Further there were significant two-way interactions of the AQ both with subscale [*F*_(9, 25, 400)_ = 2.73, *p* = 0.003] and with cue-presence [*F*_(1, 25, 400)_ = 7.01, *p* = 0.008], but no significant three-way interaction [*F*_(9, 25, 400)_ = 1.53, *p* = 0.132]. A descriptive analysis of the data suggests that participants with a higher AQ are more sensitive to the manipulation with a stronger decrease in ratings for the C− robots as compared to the C+ robot (see, Figure 4).

An exploration on the effect of prior experience with robots on participant's perception of the robots was done by Nikolovska and colleagues (2024), suggesting that participants more experienced with robots rate the social C+ robot more favorable as compared to the non-social C− robot.

## 4 Discussion

The key question driving our study was whether the previous observed over-generalization of attributions from present to absent cues (which referred to as the Pars-Pro-Toto account) can also be demonstrated for the social situations that we employed in the present study. Namely, we manipulated social sensitivity (Experiment 1), attention sharing (Experiment 2) and helping (Experiment 3). This was done by having participants rate small non-humanoid, vehicular-like robots in regard to the behavioral characteristics of interest as well as on established self-concept related questionnaires. We manipulated the robot's behavior to suggest either the presence (C+ or critical) or absence (C− or control) of one of the potential core characteristics per experiment.

### 4.1 Summary and interpretation of results

Two outcomes of the first experiment are noteworthy. First, in line with our previous results, participants over-generalized self-related ratings beyond the manipulated characteristic; they rated the critical robot higher on all characteristics except speed, equifinality, and efficiency, indicating that they perceived the C+ robot as exhibiting causal behavior, learning, context and social sensitivity, as well as exhibiting attention sharing and helping behavior. The control robot, in turn, was perceived as moving with more human-like speed and as exhibiting more goal-directed and efficient behavior. Second, participants rated the critical robot higher on most of the selfhood-attribution scales, although this did not include agency. In general, the data support our previous finding that the presence of a single behavioral cue results in the greater attribution of selfhood to the agent exhibiting a critical cue in addition to activating representations of characteristics that are missing behavioral cues in a pattern similar to what has been described as the Halo effect (e.g., Forgas and Laham, 2016).

In the second experiment, participants again rated the critical robot higher on more than one behavioral characteristic, though this time this was limited to learning and context sensitivity for the non-social characteristics, and attention sharing and helping for the social ones. While the generalization of characteristics was more limited than in Experiment 1, it still extended beyond the manipulated characteristic. Regarding the selfhood-attribution the C+ robot was rated significantly higher on all subscales except agency and safety. Hence, we replicated the observation from the first experiment that critical and control robot were not perceived to differ in their agency. While the literature suggests the importance of social factors in the attribution of agency (e.g., Carrier et al., 2014; Khamitov et al., 2016; van der Woerdt and Haselager, 2019) there are barely any studies directly investigating the effect of mere sociality. However, there is some literature on the complex link between the perception of sociality and warmth to the attribution of agency, which seems to be modulated by valence (see Suitner and Maass, 2008).

In the third experiment, participants again rated the critical robot higher on a majority of the (non-manipulated) characteristics including all social characteristics (social sensitivity, attention sharing and helping) as well as causality, equifinality, and context sensitivity. Regarding the selfhood-attribution, the C+ robot was also rated higher on all subscales except discomfort—which is the only negatively poled subscale. Overall, this indicates that participants perceived the critical robot not only as more helping, but also as more causal, goal-directed and context sensitive, as well as more socially sensitive and exhibiting more attention sharing. Participants further had the stronger tendency to attribute a self to the critical robot, unlike in Experiment 1 and 2 this effect was also observed for the subscale agency.

Across experiments, the exploratory analysis shows that, in line with the general literature (e.g., Castelli et al., 2002; Klin et al., 2003), participants with a higher indication for autism attribute less selfhood to the robots (i.e., they show a stronger tendency to take them at face-value). Interestingly, individuals with an indication for autism exhibited a stronger difference between the two conditions, implicating that they were more sensitive to the presence of social behavior than individuals with low indication for autism. Moreover, the additional analysis of the impact of people's prior experience with robots done by Nikolovska and colleagues (2024) reveals that people who are more knowledgeable about robots also were more sensitive to the manipulation of sociality in the robots. Overall, this evidence is in favor of our assumption that in attributing selfhood to other agents people refer to a subjective concept which does differ between individuals.

### 4.2 Theoretical implications

Interestingly for our purposes, the impact of manipulating these social characteristics was not restricted to the corresponding scale. More specifically, our manipulations also induced two other kinds of effect. First, and particularly important for our present purposes, the higher scores for the manipulated characteristics over-generalized to the other social scales. That is, agents showing socially sensitive behavior are also assumed to share attention with or help others, even in the absence of any perceptual evidence of such behavior, and the same kind of over-generalization was found for manipulations of social attention and helping. In other words, agents that are seen to engage in one kind of social behavior are also assumed to engage in other kinds of social behavior, even in the absence of any supporting data. This might be taken to reflect the attribution of some kind of social “personality,” of which the observed social behavior is only one example.

Second, however, the observed over-generalization was not restricted to other social characteristics. Interestingly, this kind of over-generalization could go two ways. On the one hand, some of the non-social scales showed higher ratings for the robot engaging in social activities. For instance, the socially sensitive C+ in the first experiment was also considered to show more causal behavior, learning, and context sensitivity, and similar patterns were obtained for the agents showing social attention and helping. On the other hand, however, engaging in social behavior also led to a perceived decrease with respect to other non-social characteristics. For instance, the socially sensitive robot in Experiment 1 was perceived as less oriented on the same goal (equifinality) than the control robot, which suggests some kind of trade-off between individual goal pursuit and social responsiveness.

Indeed, the way we manipulated social sensitivity (e.g., by having the critical robot stop its own navigation and turning to the other robot) reflects this trade-off. While one may consider that an experimental flaw, we take it as a valid reflection of a conceptual trade-off, in the sense that the sociality of one's behavior is defined by the degree to which one is willing to neglect one's own goal pursuit to the benefit of others. Accordingly, we consider the negative effects of engaging in social behavior on scales assessing non-social characteristics as indications of a conceptual relationship between sociality and individualism, and the respective concepts. The perhaps strongest indication of this kind of individual-social trade-off relates to agency. Whereas our previous manipulations of non-social selfhood characteristics almost always had a very strong (positive) impact on agency, this agency advantage was absent in two of our three experiments. Hence, the manipulation of social behavior has a much lesser impact on the attribution of agency than the manipulation of non-social characteristics. A similar trade-off can be found in the phenomenon described as Bystander effect (e.g., Fischer et al., 2011), which shows that being or merely imagining to be in a social situation reduces people's willingness to help (Garcia et al., 2002). The reasoning here is, that being in a group creates a diffusion of responsibility—which has been linked to a reduced sense of agency (Beyer et al., 2017). We hypothesize that this first hand experience of a disconnect between one's own actions and their outcomes, when being in a group, may lead to also perceiving other agents as having less individual agency given that they are part of a group.

Observing that people differ in how they attribute selfhood to other agents is in line with our assumption of naïve concepts of selfhood, that is, we investigated what individuals' idea of what the self is, rather than an objective definition. Considering that even in the scientific debate there are many different concepts of selfhood, it is even less surprising that in everyday life different people have different notions of what the self is. We provide evidence for a systematic link between how this selfhood-concept is attributed and, both, dispositional factors such as autism as well as more situational factors such as familiarity with the perceived agent.

Overall, the naïve approach to selfhood-attribution and the Pars-Pro-Toto account offer a novel perspective on investigating selfhood. While many theories of mind perception commonly either have a narrow focus [e.g., on agency and experience, Gray et al. (2007)] or are concerned with inferring specific mental states such as intentions (e.g., Theory of Mind, Frith and Frith, 2005; and Intentional Stance, Dennett, 1989) the Pars-Pro-Toto account takes a naïve approach that takes into account that people may differ in their ideas of what the self is. Thus, we bridge concepts like the “mind,” agency, consciousness, higher cognitive functions and sociality by considering selfhood as an umbrella term (as shown in Figure 1). Critically, our studies show, that people tend to start attributing their concept of selfhood when they perceive an agent exhibiting behavioral cues for characteristics deemed relevant to selfhood in the literature. Moreover, people seem to frequently over-generalize from cues for any single characteristic to others. Minimal information, thus, seems sufficient to activate the *entire* selfhood concept, even when other cues are absent. While this is reminiscent of the Halo-effect, our account describes an internal conceptual over-generalization rather than an evaluative bias reflecting correlational misattribution across traits.

### 4.3 Practical implications

Our findings stress the general tendency of humans to anthropomorphize agentic systems. While there is not a large body of literature investigating the movement of robots, a few examples suggest that even robot arms when moving in a human-like manner elicit anthropomorphization (e.g., Hostettler et al., 2023; Kupferberg et al., 2012). Our study extends on these findings suggesting that people even anthropomorphize small vehicle-like robots when they move in way that suggests social characteristics like sensitivity to others, attention sharing, and helping behavior; or as shown in our previous study (Pohl et al., 2024, under review) also for non-social characteristics like efficiency or learning sensitivity. Thus, anthropomorphism seems less a product of technological sophistication than of human cognitive tendencies. A possible explanation given by Urquiza-Haas and Kotrschal's (2015) is a *motor matching mechanism* inspired by (embodied) simulation theory (Goldman, 2006; Svensson et al., 2008). They argue that the mirror neuron system responsible for simulating others' actions is less dependent on the physical body of the observed agent but sensitive to actions familiar to the observer (see Buccino et al., 2004). Following, a robot of any shape navigating the physical world may trigger an observer's simulation of doing the same, thus driving anthropomorphization.

Considering the advance of technological systems into society the question is raised about moral status of artificial agents. Robots may be perceived as moral agents based on perceived rather than actual capacities, leading to misplaced moral consideration for machines and potentially creating conflicts when interacting in the same physical space (see, e.g., the perceptual believe problem, Thellman and Ziemke, 2021). This highlights the need for technological systems, especially embodied systems found in human spaces, to be designed carefully with “the right amount of anthropomorphism.” On the one side it is desirable that human-likeness is attributed, as this drives trust (e.g., Natarajan and Gombolay, 2020), on the other side, it may foster misconceptions about a system's moral status and cognitive abilities. Moreover, if selfhood attributions emerge from an observer's social expectations rather than ontological realities, then debates on robot rights or moral status may need to shift from asking whether machines possess a self to how human practices of attribution shape the moral and legal landscapes^2^.

### 4.4 Conclusion

Taken together, our study sheds light on the contribution of social factors to attributing selfhood to other agents. We showed that simple social and behavioral cues related to social sensitivity, attention sharing, and helping are relevant for attributing selfhood. We replicated our previous finding that people tend to over-generalize from one manipulated cue to other, non-manipulated cues. However, this over-generalization was more systematic within the social domain, in the sense that people tend to generalize from observed to unobserved social behavior, whereas the generalization to non-social characteristics sometimes follows other rules. With respect to some cues, this over-generalization works as within the social domain, in the sense that engaging in social behavior is assumed to imply other non-social characteristics. With respect to other cues, however, this relationship is negative, in that engaging more in social behavior reduces the perception of non-social characteristics. Further, we observed that people did not consistently attribute more agency to the social robot which may reflect the conceptual basis of how we construe sociality vis-à-vis individual goal pursuit.

## Data Availability

The datasets presented in this study can be found in online repositories. The names of the repository/repositories and accession number(s) can be found at: https://osf.io/cqf6d/.

## Footnotes

^1^Note that the manipulation check scale includes cues that were not directly related to the experiments reported here, as this paper is a follow-up study extending the manipulation to social characteristics.

^2^See also the paper by (Gunkel 2018), for an in-depth discussion of this shift toward an approach of seeing robots in relation to humans, rather than looking at robots' capabilities.
